# Characteristics of Saliva in Syphilitics

**Published:** 1882-05

**Authors:** A. W. Harlan


### CHARACTERISTICS OF SALIVA IN SYPHILITICS.

Not very much will be said concerning the saliva of persons infected with the virus of syphilis, for in most cases there is no appreciable difference existing between such saliva and that from the mouth of a perfectly healthy subject. It is not my purpose to dwell on the characteristics of syphilitic saliva, for the reason that they have no practical bearing in connection with the practice of dentistry except as shall be mentioned further along, but the lesions of the mouth, resulting from inherited and acquired syphilis, are so numerous in form and variety and are so often destructive to the bones of the nose and mouth, that it is incumbent on all dental surgeons to be familiar with their appearance, duration and treatment, primarily for the benefit of the patient, and secondarily for self-protection. Therefore it has been suggested that a brief resumé of the order, character and general appearance of mouth lesions may not prove uninteresting. Saliva from the mouth of a person suffering with syphilis in its second stage presents only this marked physical difference from saliva in general : a sufficient quantity collected for the purpose is poured into a vessel containing water, it sinks more quickly and does not mingle with the water so readily as non-syphilitic saliva ; on testing, it is found to contain more albuminous matter, and when there are extensive patches and erosions of the mouth and pharynx it is secreted in abundance, accompanied by increase in size of the submaxillary glands ; during the eruptive period of syphilis it is very much lessened in volume and gives an acid reaction. Saliva is the only one of the physiological secretions which is apt to be the vehicle in spreading the poison of syphilis, examples of which are here presented.

In one case a sailor went from house to house tattooing, and was observed to wet the needles in his mouth before pricking in the colors. Fifteen out of twenty-two persons tattooed acquired syphilis. On examination his mouth and throat were found covered with ulcers and erosions ; the pus mingling with the saliva being pricked in with the coloring matter caused the inoculation. Two persons were indiscreet enough to use a tooth-brush which had previously been used by a syphilitic; they both acquired the disease. An old Frenchman smoked a pipe which had been used only a short time previously by a syphilitic friend, and in due course the initial chancre appeared on his lip. By searching the literature of syphilis, numerous cases may be found where kissing has been the means of communicating the disease. Two cases are reported by Keyes. In twelve thousand cases reported from hospitals, three and one third per cent exhibited the primary sore on the lip or within the mouth; more than ninety four per cent of the remainder showed the chancre on the genitals or anus. Whistles and trumpets sold on the streets and wet with the saliva of the vendors may be infectious. In some regions where glass-blowing establishments are numerous, laws have been enacted to prevent the use of the mouth tubes except by the owners, on account of the liability of communicating syphilis. Any article used by a syphilitic in the mouth when it is eroded or covered with ulcerous patches may inoculate the innocent user, if it be not thoroughly cleansed. Many an innocent nurse has acquired syphilis by suckling a child inheriting it, as the mouth lesions appear so early in most cases that they are unable to escape unless the nipple remains unabraded. There are some who believe that the twisted end of a new cigar having been wet with saliva of a syphilitic may be contagious. The use of the rubber dam in the mouth of a syphilitic, and its subsequent use in the mouth of another, without perfect cleansing, might prove infectious; it would be if any blood were adherent, as it is known to be infectious. Forceps that have been used and not thoroughly cleansed, scalers and lancets, particularly abscess lancets, and other instruments used by dentists, oculists, and aurists, unless properly cleansed, would spread syphilis. In circumcision and vaccination numerous cases are reported, especially in the latter. In transplanting teeth it would be possible to communicate syphilis, but J. Hunter's claim that because an abscess formed opposite the roof of the transplanted tooth in three or four weeks after the operation, that it was a chancre, is of course erroneous; the abscess appeared because the pulp was not removed from the tooth, and in three or more weeks it decomposed and the gas had to have exit; hence the abscess. It is impossible to point out the numerous methods of acquiring syphilis in a brief paper. Suffice it to say that we cannot be too careful of our own fingers or our patients' mouths in any suspected case.

An abrasion or a surface capable of absorption, if it have, in contact with it, the poison of syphilis for a sufficient period of time, no matter where the spot be located, is as sure to exhibit the chancre as it is sure to result from sexual contact when one of the persons is diseased. From the fact that persons who acquire syphilis usually resort to the venereal specialist, dentists generally may be ignorant of the cause of the disease in its primary form, except from reading or when medically educated, but when the secondary symptoms progress far enough to exhibit excoriations, erosions, ulcers and mucous patches in the mouth and throat, the dermatologist necessarily sends his patients to a dentist in order that all tartar shall be removed from the teeth, rough corners filed off and pieces of roots removed, ill fitting dentures trimmed and polished, so that the tongue, cheeks and lips may be free from such sources of irritation. If the person infected falls into the hands of a dentist before consulting a physician, his knowledge of the clinical features of the disease ought to enable him to attend to the hygiene of the mouth so effectually that the treatment of the case constitutionally may be left to the venereal specialist until a cure is effected.

An outline may now be presented of the course of syphilis. After contact with the poison a chancre appears in three or four weeks, or longer; the chancre always appears at the point of inoculation; in two weeks thereafter the adjacent lymphatic glands enlarge, but seldom suppurate. After a month or six weeks has elapsed, an eruption on the skin, which may be quite general, appears, usually preceded by slight fever, accompanied by headache, rheumatic pains at night, and an enlargement of the epi-trochlear and post cervical glands; coincident with these symptoms the mouth lesions are met with, consisting of mucous patches, erosions and ulcers; they are concomitant from time to time with other general symptoms of the disease during its whole course. After the expiration of a year, the mouth lesions are much worse, being usually whitened and excoriated patches, ragged ulcers upon the fauces and in the mouth, especially deep ulcers on the inside of the lower lip and opposite the last molar teeth. The tongue white and furred, which cannot be scraped off without bleeding, its edges and under surface raw and the adjacent nasal mucous membrane covered with dark yellow and brownish scabs, a thickening and fissuring of the orifice of the nostrils, with the breath quite offensive. The mucous patch when once seen is not likely to be confounded with that from any other affection. It is nearly round and raised slightly, of a dirty white color, sometimes red or granulated, and covered with a puriform secretion : its size varies from a point to that of a large copper cent, patches occur on the tonsils, the whole of the pharynx, within the lips, the nose, trachea, larynx, and upon the tongue or under it. They are generally painless unless surrounded by erythema, irritated by smoking, or a rough tooth, or some similar cause. A person with a mucous patch upon the lip or within it, is far more dangerous to a community than half a dozen people with developed chancres on the genitals, as may readily be seen when it is known that the secretion from it is infectious. Chancre of the lip, which is most often seen in hospitals, is globular or oval, angry looking and the size of a nickel ; it is highly infectious. Scaly patches appear in the mouth after two or more years, and their favorite locality is at the angle of the mouth, the tip, sides and dorsum of the tongue ; frequently masses of overgrowth with adherent epithelium cover a patch under the tongue, which, when detached, are as thick as a knife blade ; in some respects they resemble icthyosis, but a close inspection of this syphilide marks its color a mottled bluish white, and no mistake need be made concerning its origin ; these usually require nothing more than local treatment. Gummy tumors of the hard and soft palate come without pain, but when once formed they destroy all tissue which has become infiltrated ; they are peculiarly destructive of the bones of the mouth and nose, and need to be heroically treated with the iodides or mercury, internally.

It is generally agreed that syphilis causes the most extensive mouth and throat ravages, especially destructive of bone, when no previous scrofula or lupus can be traced, hence the disfigured nasal organs and injuries of the hard and soft palate, which are frequently irreparable. Gumma of the tongue differs from epithelioma in this : it occurs at any age, has syphilitic history, commences deep and feels like a pea between the fingers, is sometimes multiple, and *may always be found on the posterior aspect and sides, but never underneath the tongue.* When ulceration takes place, it uncovers a deep cavity and is usually painless, which is characteristic. When excised completely, it does not recur. The appearance of mouth lesions from inherited syphilis is often as early as a week from birth to a few months or even years, yet the latter occurs very rarely. Snuffling, and a general coryza, with excoriations around the nose and mouth, indicate very plainly the cause of the trouble ; the child whines, its voice is cracked, and the skin drawn tightly over the face, which gives it an oldish look, but the development of patches at the angles of the lips and orifices of the nostrils are usual and correct points in diagnosis. After the seventh year the notched superior central incisors are a sure indication of inherited syphilis. The whole train of symptoms are observed from inherited that are seen in acquired syphilis, except that there is no chancre ; the lymphatic glands do not enlarge, and the nails are affected either by impairment of nutrition or ulcerative onychia, the latter of which is most frequently noticed.

The local treatment of mouth lesions from acquired or inherited syphilis, is perfect cleanliness, the use of a soft brush and an alkaline powder or wash ; the teeth should be polished and roots be extracted or pivoted ; tobacco must be forbidden ; and mild astringent washes and gargles be prescribed for young children. Strong warm tea with X grs. borax to the ℥i, or V grs. chlorate of potash to the same, are effectual for infants and children. Patches and ulcers may be painted with a solution of nitrate of silver, or solution of corrosive chloride of mercury, grs. iij to ℥i alcohol, daily ; or when necessary they should be touched lightly and carefully with a solution of acid nitrate of mercury twice a week, or any other favorite effectual remedy may be used. If there are gummata likely to involve the bone, iodide of potassium or iodide of sodium should be prescribed in V to X gr. doses or larger, during or after meals. The sodium causes less disturbance of the stomach. If the bones of the mouth have become denuded of their periosteum, no effort should be spared to prevent further caries or necrosis, but no local remedy will be found efficient. If the dentist be timid or wanting in definite knowledge of the needs of the patient, he should be sent to his physician without loss of time. In most cases the dead bone will separate from the living during prompt and appropriate constitutional treatment. Caries must be operated upon beyond the line of decay, then pursue a systematic treatment, which, if kept up sufficiently long, will certainly result in a cure.—*Dr. A. W. Harlan, Transactions Illinois State Dental Society.*

